# Targeting the inward rectifier potassium channel 5.1 in thyroid cancer: artificial intelligence-facilitated molecular docking for drug discovery

**DOI:** 10.1186/s12902-023-01360-z

**Published:** 2023-05-19

**Authors:** Xue Yang, Yonglin Wu, Shaojie Xu, Hanning Li, Chengcheng Peng, Xiaoqing Cui, Deenraj Kush Dhoomun, Ge Wang, Tao Xu, Menglu Dong, Xingrui Li, Yaying Du

**Affiliations:** 1 Department of Thyroid and Breast Surgery, Tongji Hospital, Tongji Medical College, Huazhong University of Science and Technology, Wuhan, Hubei People’s Republic of China; 2 Laboratory of Thyroid and Breast Surgery, Tongji Hospital, Tongji Medical College, Huazhong University of Science and Technology, Wuhan, Hubei People’s Republic of China; 3 Laboratory of General Surgery, Tongji Hospital, Tongji Medical College, Huazhong University of Science and Technology, Wuhan, Hubei People’s Republic of China; 4 Department of Thyroid and Breast Surgery, Huanggang Central Hospital, Huanggang, Hubei People’s Republic of China; 5 Department of Obstetrics and Gynecology, Cancer Biology research center, Tongji Hospital, Tongji Medical College, Huazhong University of Science and Technology, Wuhan, Hubei People’s Republic of China

**Keywords:** Differentiated papillary thyroid cancer, Thyroid-stimulating hormone receptor, Artificial intelligence, Deep docking, Computer-aided drug discovery, KCNJ16/Kir5.1

## Abstract

**Background:**

Recurrent and metastatic thyroid cancer is more invasive and can transform to dedifferentiated thyroid cancer, thus leading to a severe decline in the 10-year survival. The thyroid-stimulating hormone receptor (TSHR) plays an important role in differentiation process. We aim to find a therapeutic target in redifferentiation strategies for thyroid cancer.

**Methods:**

Our study integrated the differentially expressed genes acquired from the Gene Expression Omnibus database by comparing TSHR expression levels in the Cancer Genome Atlas database. We conducted functional enrichment analysis and verified the expression of these genes by RT-PCR in 68 pairs of thyroid tumor and paratumor tissues. Artificial intelligence-enabled virtual screening was combined with the VirtualFlow platform for deep docking.

**Results:**

We identified five genes (KCNJ16, SLC26A4, TG, TPO, and SYT1) as potential cancer treatment targets. TSHR and KCNJ16 were downregulated in the thyroid tumor tissues, compared with paired normal tissues. In addition, KCNJ16 was lower in the vascular/capsular invasion group. Enrichment analyses revealed that KCNJ16 may play a significant role in cell growth and differentiation. The inward rectifier potassium channel 5.1 (Kir5.1, encoded by KCNJ16) emerged as an interesting target in thyroid cancer. Artificial intelligence-facilitated molecular docking identified Z2087256678_2, Z2211139111_1, Z2211139111_2, and PV-000592319198_1 (-7.3 kcal/mol) as the most potent commercially available molecular targeting Kir5.1.

**Conclusion:**

This study may provide greater insights into the differentiation features associated with TSHR expression in thyroid cancer, and Kir5.1 may be a potential therapeutic target in the redifferentiation strategies for recurrent and metastatic thyroid cancer.

**Supplementary Information:**

The online version contains supplementary material available at 10.1186/s12902-023-01360-z.

## Introduction

Thyroid cancer is projected to become the fourth leading type of cancer with a substantial rise in its global incidence [1]. According to the histological classification, follicular thyroid cell-derived tumors can be classified as papillary thyroid carcinoma (PTC), follicular thyroid carcinoma (FTC), poorly differentiated thyroid carcinoma, and anaplastic thyroid carcinoma (ATC) [2]. Approximately 30% of the well-differentiated thyroid cancers (WDTC) progress to dedifferentiated thyroid cancers (DeTCs) and develop recurrence and metastasis [3]. Hence, the dedifferentiation status of DeTC and ATC indicates a more invasive approach and worse prognosis. Radioactive iodine (RAI) is the dominant therapy for WDTC patients with recurrent and metastatic diseases. The loss of differentiation features represents the reduced expression of thyroid-specific genes, such as the thyroid-stimulating hormone (TSH) receptor (TSHR), sodium/iodide symporter (NIS), thyroglobulin, thyroperoxidase (TPO), and paired box transcription factor 8 [4]. Such patients display resistance to RAI therapy (RAIR) and cannot benefit from RAI treatment. Thus, restoring differentiation may be a promising strategy for overcoming cancer invasion and RAIR.

TSHR is a representative thyroid-specific gene that reveals the differentiation status of the thyroid cells. The expression of TSHR consistently induces the formation of colonies with epithelial morphology and thyroglobulin expression [5]. TSH-TSHR signaling induces thyrocyte proliferation and the expression of other thyroid-specific genes. TSHR is required for optimal NIS expression and its localization to the plasma membrane, thus ensuring a response to RAI therapy [6, 7]. In the absence of functional TSHR, the thyroid gland develops to a normal size, whereas the expression of thyroperoxidase and sodium/iodide symporter gets significantly reduced [8]. Evidence from numerous studies on the resected thyroid tissue suggest that TSHR is more persistently expressed than other differentiation markers (including NIS and thyroglobulin ), thereby indicating it is a prominent differentiation marker [9].

As a marker of thyroid differentiation, TSHR expression may affect the function of cancer cells that have undergone malignant transformation, such as epithelial-mesenchymal transition (EMT) and dedifferentiation. Promoting TSHR expression may facilitate the transformation of DeTC to WDTC. Taken together, we analyzed the Cancer Genome Atlas (TCGA) database and Gene Expression Omnibus (GEO) database, grouped by TSHR expression level, screened WDTC and ATC, finally identified two differentially expressed genes (DEGs), namely KCNJ16 and SYT1. Subsequently, we verified the expression change of TSHR, KCNJ16 and SYT1 in 68 pairs of normal and tumor tissues. During exploration, we found the significant role of KCNJ16 in thyroid differentiation and cancer associated signaling pathways. Therefore, we targeted the protein encoded by KCNJ16 and used artificial intelligence facilitated molecular docking to search potential molecular for drug discovery.

## Materials and methods

### Patients sample collection

Seventy pairs of primary tumors and adjacent normal tissues were collected from patients diagnosed with thyroid tumor between January 2021 and September 2021 at the affiliated Tongji Hospital of Tongji Medical College. We recorded their clinical and pathological information, including demographics, historical types, metastasis and invasion status, BRAF V600E status, and tumor stage. Of the 70 patients, six, 36, and 26 patients were diagnosed with benign thyroid tumors, PTC, and papillary thyroid microcarcinoma (PTMC). Moreover, two patients had medullary thyroid carcinoma (MTC). We excluded these patients from the subsequent analysis because the sample size was considerably small and MTC lacked TSHR expression. All patients signed an informed consent form. All methods were approved by the Research Ethics Committee of the Tongji Medical College, and this study was conducted in accordance with declaration of Helsinki.

### Gene expression profiles and data preprocessing

Transcriptomic data of WDTC were collected from TCGA database. We downloaded HTseq-counts of 510 patients with differentiated thyroid carcinomas (DTC) from TCGA website [10], which were ordered by the TSHR mRNA expression levels. We classified 100 samples of the lowest TSHR expression into the “low-TSHR” group; contrarily, 100 samples of the highest TSHR expression were classified into the “high-TSHR” group. Eventually, we identified significant DEGs using the EdgeR package in R (The R Project for Statistical Computing). DEGs related to KCNJ16 were available by comparing the “high-KCNJ16” and “low-KCNJ16” group. The dividing line represented the median expression level of KCNJ16. The criteria were set as P = 0.05 and |log2 fold change (FC)| = 2.

We obtained RNA sequencing data of ATC from the National Center for Biotechnology Information GEO. mRNA expression data were extracted and merged from GSE29265, GSE33630, GSE65144, and GSE76039 (Additional Table 1). We performed normalization and batch-normalization using an Sva package of the R Project. Considering the data scale, we selected 10 samples of the lowest and highest expression of TSHR mRNA as the “low-TSHR” and “high-TSHR” groups, respectively. DEGs were identified using the Limma package, which processed the data of both groups. We used criteria similar to those described above. Gene Ontology (GO), Kyoto Encyclopedia of Genes and Genomes (KEGG) and Gene Set Enrichment Analyses (GSEA) of DEGs were performed using the clusterProfiler package of the R Project. The threshold for statistical significance was set at a P-value of 0.05.

In addition, we performed a gene expression profiling interactive analysis (GEPIA) [11], and used cBioPortal for Cancer Genomics [12] to determine the correlation between the two targeted genes. Gene expression in different phenotypes of thyroid cancer was from UALCAN. TIMER and GSCA database were used to analyze immune infiltration, Survival curve of target genes in thyroid cancer was from Kaplan-Meier Plotter. MEXPRESS supplied analysis of DNA methylation status.

### Total RNA extraction and real-time polymerase chain reaction

Fresh surgical specimens were immersed in RANwait (Biosharp, Anhui, China), a storage reagent to protect RNA from degradation. Total RNA from thyroid tissues was extracted using the TRIzol reagent (CWBIO, Jiangsu, China) according to the manufacturer’s instructions. We converted RNA (2ug) to cDNA using the Hifair III 1st Strand cDNA Synthesis SuperMix for quantitative PCR (qPCR) (YEASEN, Shanghai, China). Real-time PCR was performed using the ChamQ Universal SYBR qPCR Master Mix (Vazyme, Nanjing, China). The primers were synthesized by Tsingke (Beijing, China; Additional Table 1). Upon pairing the tumor tissues and their adjacent paratumor tissues from different patients, we calculated the 2^−ΔCt^ (ΔCT = CT_(target gene)_ - CT_(GAPDH)_) of the target genes to compare the expression levels of the tumor tissues and paired normal tissues. Quantitative data were obtained from three replicate experiments.

### Construction of the plasmid and stable transfected cell lines

The nucleotide sequence of the KCNJ16-CDS was extracted from the CCDS database [13]. The CDS of KCNJ16 was provided in Additional File 1. KCNJ16-CDS was inserted into the over-expression vector PLVX-EF1α-IRES. The lentiviral vector was packaged using the VSVG and Gag plasmids. PTC cell line K1 and FTC cell line FTC133 were transfected with the lentivirus, following which the stably transfected cell lines were screened using puromycin.

### Artificial intelligence-enabled deep docking

The REAL library of enamine can be accessed through a graphical interface [14]. We used the DD docking protocol as described in part A [15] and narrowed down the library running VirtualFlow for the final docking. The PDB file of Kir5.1, from the A.I. system AlphaFold, was used to predict its 3-D structure [16, 17]. We performed receptor preparation using AutoDock Vina v.1.2.0, and the grid box parameters were set as the x center = 25.073, y center = 15.936, and z center = -41.275. The results were visualized using PyMOL v2.5.3. The programs were used with the majority of the default docking parameters. Runtime environment: ubuntu 20.04; slurm; open shserver.

### Statistical analysis

Statistical calculations and the visualization of gene expression were performed using GraphPad Prism (Version8.0.2). Initially, we performed normality and lognormality tests. We performed paired t-tests to compare targeted gene expression in the tumor tissues and corresponding normal tissues. Spearman’s correlation and linear regression analyses depicted the associations between the two genes. To describe the distribution of TSHR and KCNJ16 expression in different clinicopathological features, we performed the Student’s t-test and Mann-Whitney test for two groups of normally distributed variables and non-normally distributed variables, respectively. The Kruskal-Wallis test was performed to compare the multigroup data. Statistical significance was set at P < 0.05.

## Results

### DEGs screened by the TSHR expression level

We analyzed TSHR expression in the clinicopathological features of thyroid cancer (Table 1). The age and invasion status, including the capsular, vascular, and extrathyroidal extensions, were closely related to TSHR expression in tumor tissues. Patients aged ≥ 55 years and those with cancer extension revealed decreased TSHR mRNA expression. TSHR, as a differentiation indicator, had varying expression in different phenotypes of thyroid cancer. The correlation between TSHR expression and the two prognostic indicators indicated the role of TSHR in thyroid cancer. Therefore, the differentially expressed genes were analyzed according to the expression level of TSHR. DEGs were compared between the “high-TSHR” and “low-TSHR” groups.

**Fig. 1:**
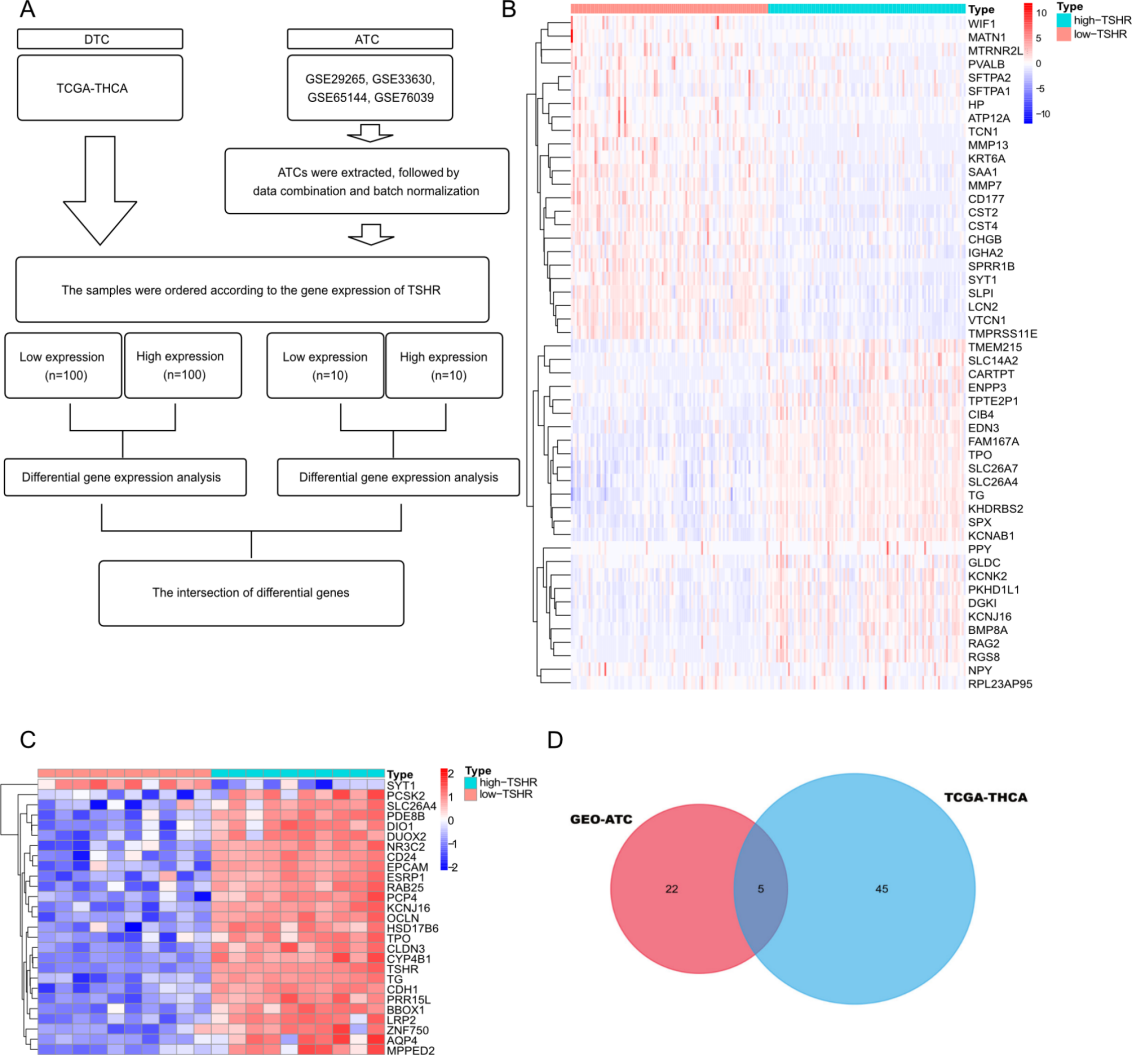
DEGs screened by the TSHR expression level (**A**) The flow chart of DEG screening process from TCGA database and GEO database. (**B**) The expression regulation of 50 DEGs between “high-TSHR” group and “low-TSHR” group in thyroid cancer patients from TCGA database. (**C**) The expression regulation of 27 DEGs between “high-TSHR” group and “low-TSHR” group in ATC patients From GEO database. (**D**) Venn diagram of the two DEGs datasets. DEG: differentially expressed gene, TSHR: thyroid stimulating hormone receptor, ATC: anaplastic thyroid cancer

**Fig. 2:**
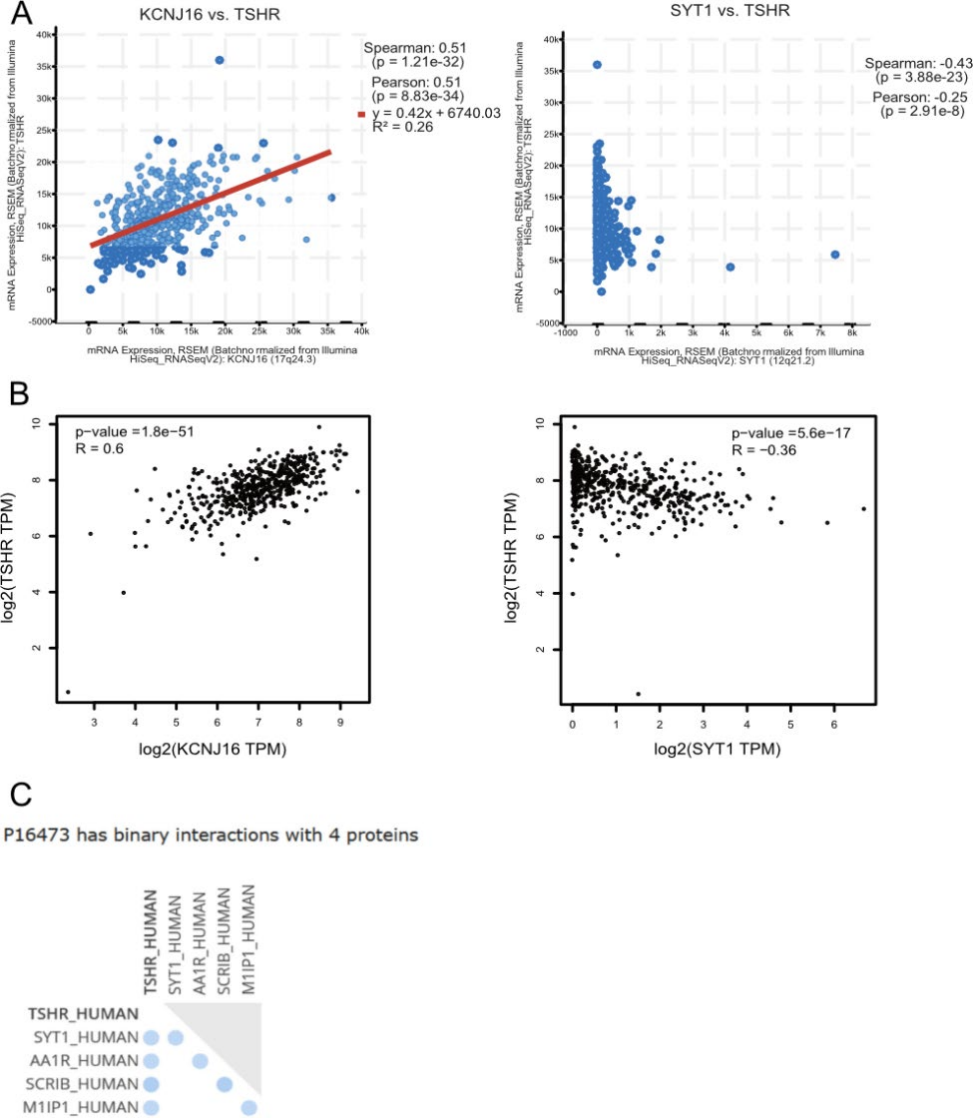
The primary analysis of correlation between TSHR and DEGs. (**A**) The correlation analysis between KCNJ16-TSHR mRNA expression and SYT1-TSHR mRNA expression on the cBioPortal website (http://www.cbioportal.org/). (**B**) The correlation analysis between KCNJ16-TSHR mRNA expression and SYT1-TSHR mRNA expression on the GEPIA website (http://gepia.cancer-pku.cn/). (**C**) The binary interaction between TSHR and SYT1 on the Uniport protein database (https://www.uniprot.org/)

Figure 1 A depicted the flow chart of the DEG screening. Data were collected from 510 patients with DTC and 52 patients with ATC from TCGA and GEO database, respectively. Finally, five genes were differentially expressed simultaneously by integrating 50 DEGs from TCGA with 27 DEGs from GEO, namely KCNJ16, SLC26A4, TG, TPO, and SYT1 (Fig. 1B and D). KCNJ16 and SYT1 were our target genes because SLC26A4, TG, and TPO were the classic genes in thyroid and the downstream of the TSH-TSHR signaling pathway [2]. As in Fig. 2, we analyzed the association between KCNJ16, SYT1, and TSHR using GEPIA and cBioPortal. The mRNA expression of KCNJ16 revealed a positive correlation with TSHR (R = 0.51, P < 0.05), which was consistent with a previous result that KCNJ16 was upregulated in the “high-TSHR” group. SYT1 expression still displayed a negative correlation with TSHR (R = -0.43, P < 0.05). Binary interactions between TSHR and SYT1 was observed in the UniProt database.

**Table 1:** Association of TSHR mRNA expression with different variables of thyroid tumor patients

Variable	Cases (n = 68(%))	TSHR expression (2^−ΔCt^)	P-value
Gender			
Female	50(74)	0.1573(0.1033, 0.2166)	0.72
Male	18(26)	0.1427(0.1288, 0.2689)	
Age/yr			
<55	56(82)	0.1609(0.1279, 0.2541)	**0.01** ^*^
≥55	12(18)	0.1121(0.0751, 0.1555)	
Tumor diameter/cm			
≤2	58(85)	0.1518(0.1115, 0.2334)	0.77
>2	10(15)	0.1450(0.1288, 0.2191)	
Histological types			
Benign	6(9)	0.1483(0.1311, 0.1956)	**0** ^*^
PTC^a^	36(53)	0.1292(0.0802, 0.1986)	
PTMC^b^	26(38)	0.1910(0.1458, 0.2721)	
Lymph node metastasis			
Yes	41(60)	0.1473(0.1115, 0.2511)	0.91
No	27(40)	0.1488(0.1275, 0.2193)	
Vascular/capsular invasion			
Yes	28(41)	0.1327(0.0776, 0.1581)	**0** ^*^
No	40(59)	0.1815(0.1393, 0.2627)	
BRAF V600E			
Wild type	21(31)	0.1392(0.1221, 0.1928)	0.34
Mutation	47(69)	0.1613(0.1217, 0.2451)	
Stage			
Stage I	57(84)	0.1613(0.1253, 0.2468)	**0** ^*^
Stage II+III	5(7)	0.0748(0.0383, 0.1043)	

a: papillary thyroid carcinoma

b: papillary thyroid microcarcinoma

*: Bold fonts indicate statistical significance.

### Expression analysis and prognostic value of DEGs in thyroid cancer

**Fig. 3:**
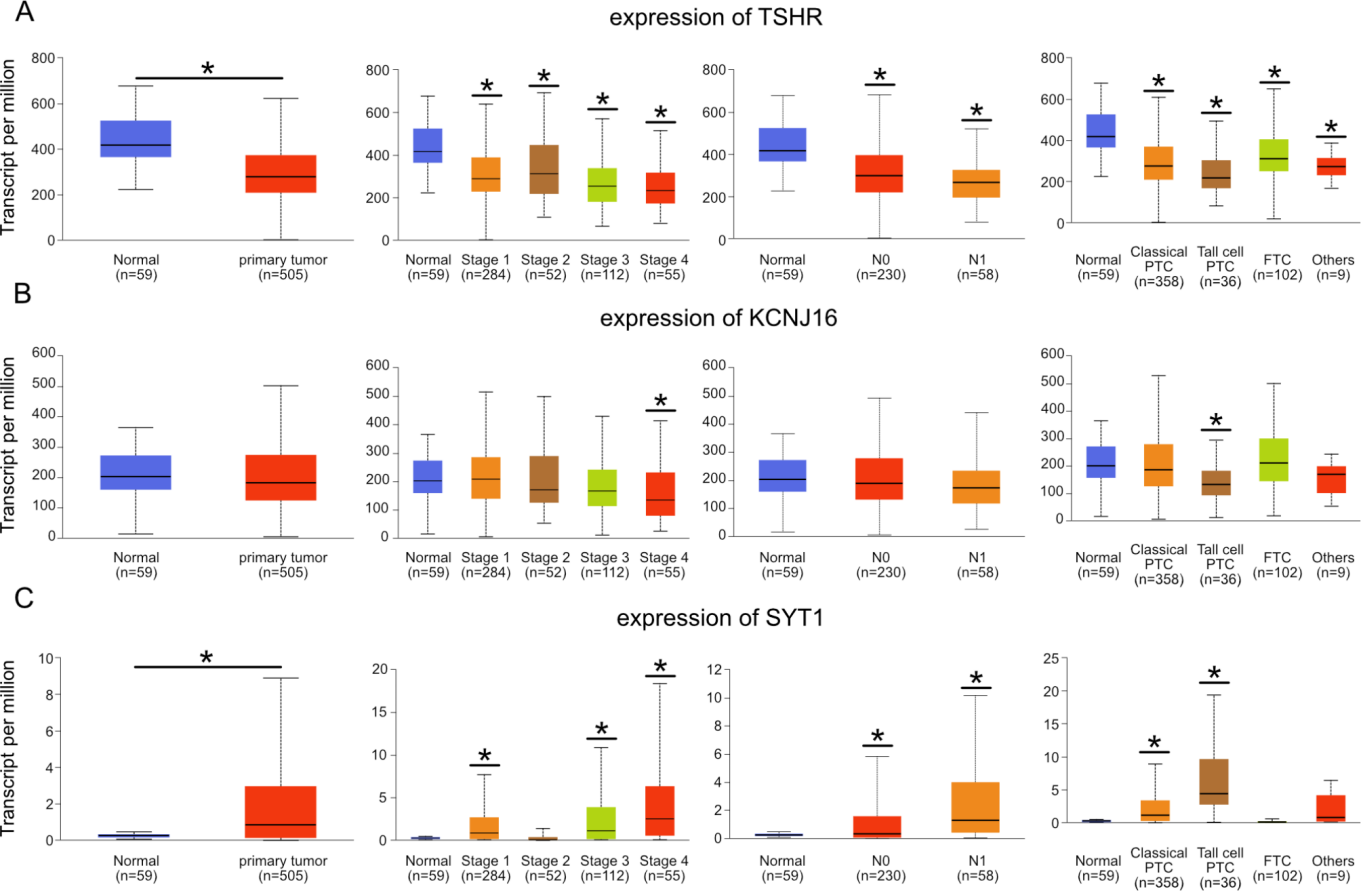
Expression of TSHR, KCNJ16 and SYT1 in thyroid cancer phenotypes from the UALCAN. (**A**) Box plots of TSHR transcripts in sample types, stages nodal metastasis status and histological subtypes of thyroid cancer from TCGA database. (**B**) Box plots of KCNJ16 transcripts in thyroid cancer. (**C**) Box plots of SYT1 transcripts in thyroid cancer. N0: No regional lymph node metastasis, N1: Metastases in 1 to 3 lymph nodes, PTC: papillary thyroid carcinoma, FTC: follicular thyroid carcinoma, *: P < 0.05

We used TCGA database in UALCAN to investigate DEGs expression in thyroid cancer. TSHR was decreased in primary tumor and in any stages or histological subtypes when compared to normal group, indicating it was a strong biomarker of thyroid cancer (Fig. 3A). The down-regulation of KCNJ16 was significant in stage 4 and tall thyroid papillary carcinoma (Fig. 3B). KCNJ16 expression change might come from DNA methylation, cause its expression in thyroid cancer was negatively correlated with DNA promoter methylation and CpG hypermethylation appeared in metastatic group and thyroid papillary carcinoma - tall cell ( > = 50% tall cell features) (Additional Fig. 1A and B). Tall cell morphology was an aggressive variant of papillary thyroid carcinoma that has been associated with poor outcomes [18]. SYT1 expression increased in primary tumor, almost in all stages when compared to normal group. And SYT1 was also observed in tall thyroid papillary carcinoma (Fig. 3C).

**Fig. 4:**
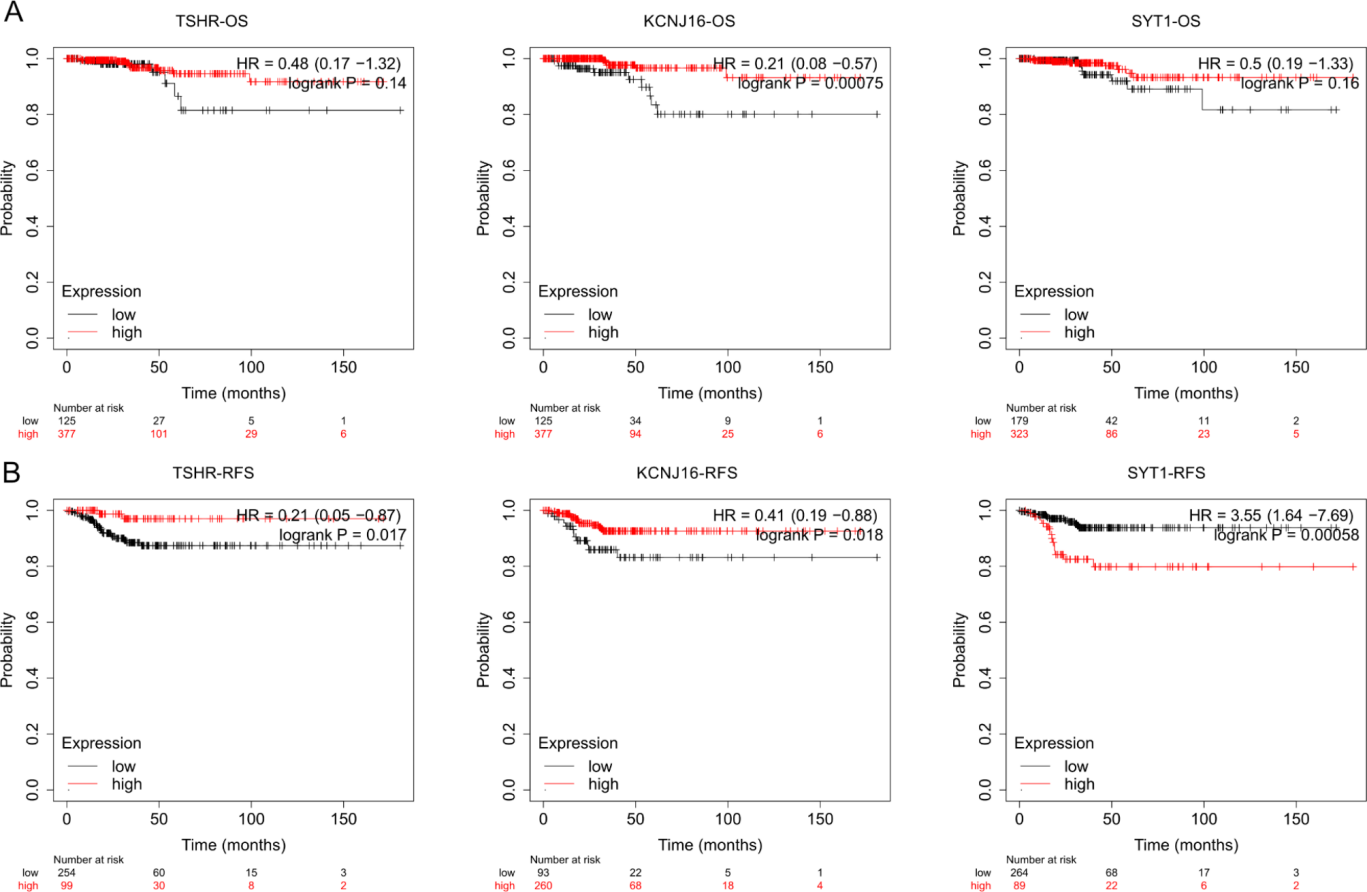
Survival curve of TSHR, KCNJ16 and SYT1 in thyroid cancer patients from Kaplan-Meier plotter (**A**) The OS probability of high expression and low expression of TSHR, KCNJ16 and SYT1 in thyroid cancer patients. (**B**) The RFS probability of high expression and low expression of TSHR, KCNJ16 and SYT1 in thyroid cancer patients. Logrank P indicates statistical significance. OS: overall survival, RFS: recurrence-free survival, HR: hazard ratio

Then we applied KM plotter to investigate their prognostic value in thyroid cancer, TIMER and GSCA to analyze immune cell infiltration and survival. From Fig. 4, high expression of TSHR (HR = 0.21, logrank P = 0.017) and low expression of SYT1 (HR = 3.55, logrank P = 0.00058) were significantly correlated with favorable RFS. KCNJ16 supported good prognosis of OS (HR = 0.21, logrank P = 0.00075) and RFS (HR = 0.41, logrank P = 0.018). The tumor immune microenvironment of thyroid cancer was composed of cancer cells, tumor-associated fibroblasts and immune cells. Immune cells have complicated roles in tumor growth, differentiation, angiogenesis and migration [19]. Changes in TSHR, KCNJ16 and SYT1 expression level were accompanied by the alteration of immune cells infiltration. But the cumulative survival curves showed that the infiltration of B cell, CD8 + T cell, CD4 + T cell, macrophage, neutrophil and dendritic cell didn’t cause a significant change in survival time of thyroid cancer patients (Additional Fig. 2A). But from the GSCA database, TSHR and KCNJ16 shared a similar correlation with immune infiltrates. And SYT1 expression was positively correlated with the infiltration of iTreg, nTreg and macrophage (Additional Fig. 2B).

### DEGs expression in clinical samples and their role in thyroid cancer

**Fig. 5:**
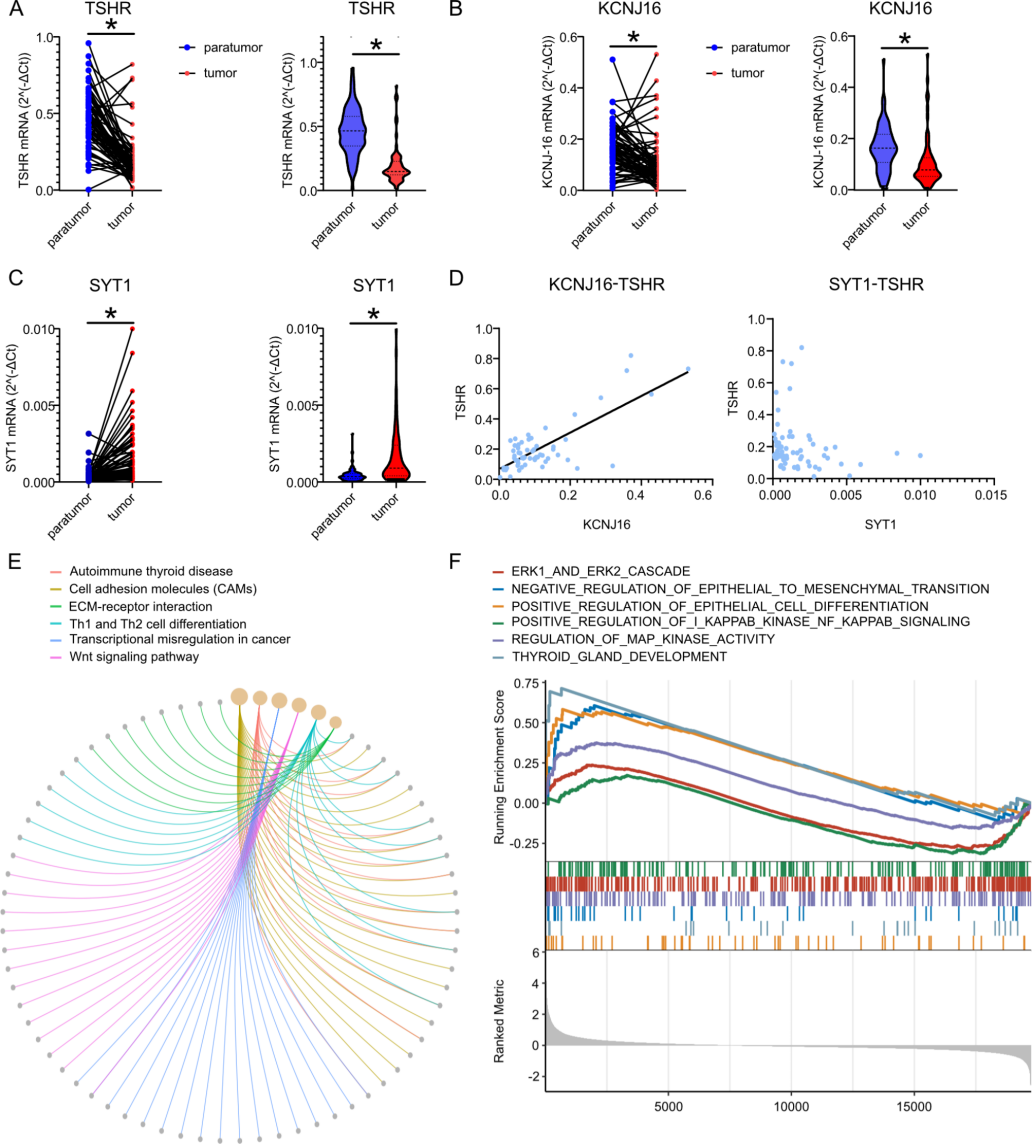
Target genes expression in clinical samples and DEGs enriched pathways (**A**) Individual expression and median expression of TSHR in paratumor and tumor tissue. (**B**) Individual expression and median expression of KCNJ16 in paratumor and tumor tissue. (**C**) Individual expression and median expression of SYT1 in paratumor and tumor tissue. (**D**) The correlation analysis of KCNJ16-TSHR expression and SYT1-TSHR expression of clinical samples. (**E**) The enriched pathways of DEGs in “high-KCNJ16” group of thyroid cancer patients from TCGA database. (**F**) The GSEA analysis of listed pathways in ATC patients from GEO database. The running enrichment score from − 0.25 to 0.75. DEG: differentially expressed gene, GSEA: gene set enrichment analysis, ATC: anaplastic thyroid cancer. *: P < 0.05

RT-qPCR experiments were performed to verify the mRNA expression levels of the identified DEGs. We analyzed mRNA expression levels in 68 thyroid tumor samples and adjacent normal tissues. The expression of TSHR and KCNJ16 was lower in the tumor tissues than that in the paired normal tissues, whereas the expression of SYT1 was upregulated in the tumor tissues (Fig. 5A C). The correlations were nearly consistent with the prior analysis. KCNJ16 mRNA expression was positively correlated with TSHR mRNA expression (Pearson r = 0.7734, P < 0.05). However, our results did not demonstrate an association between SYT1 and TSHR expression (Pearson r = 0.045, P = 0.7283) (Fig. 5D).

Subsequently, we explored KCNJ16 expression in different clinical and pathological features of thyroid cancer (Table 2). Conspicuously, KCNJ16 expression was decreased in patients with capsular and vascular invasion or an extrathyroidal extension invading the larynx, trachea, or esophagus, in the absence of significant changes among other clinicopathologic features. Simultaneously, we observed the downregulation of TSHR expression in patients with capsular/vascular invasion (Table 1). Both differences were statistically significant.

**Table 2:** Association of KCNJ16 mRNA expression with different variables of thyroid tumor patients

Variable	Cases (n = 68(%))	KCNJ16 expression (2^−ΔCt^)	P-value
Gender			
Female	50(74)	0.08296(0.05275, 0.1277)	0.56
Male	18(26)	0.06787(0.04606, 0.1108)	
Age/yr			
<55	56(82)	0.08515(0.05309, 0.1255)	0.13
≥55	12(18)	0.06295(0.01532, 0.1223)	
Tumor diameter/cm			
≤2	58(85)	0.07784(0.05204, 0.1241)	0.86
>2	10(15)	0.08093(0.05070, 0.1331)	
Histological types			
Benign	6(9)	0.08436(0.04653, 0.1111)	0.43
PTC^a^	36(53)	0.06598(0.04574, 0.1220)	
PTMC^b^	26(38)	0.08296(0.05831, 0.1414)	
Lymph node metastasis			
Yes	41(60)	0.08668(0.04913, 0.1305)	0.58
No	27(40)	0.06807(0.05294, 0.1219)	
Vascular/capsular invasion			
Yes	28(41)	0.06485(0.04078, 0.1002)	**0.02** ^*^
No	40(59)	0.08949(0.05475, 0.1527)	
BRAF V600E			
Wild type	21(31)	0.08361(0.05431, 0.1176)	0.82
Mutation	47(69)	0.07322(0.05159, 0.1303)	
Stage			
Stage I	57(84)	0.08232(0.05325, 0.1305)	0.07
Stage II+III	5(7)	0.02196(0.007871, 0.1285)	

a: papillary thyroid carcinoma

b: papillary thyroid microcarcinoma

*: Bold fonts indicate statistical significance

Considering the coherent correlation between KCNJ16 and TSHR expression and their tendency to decline in tumor tissues, we focused on the potential role of KCNJ16 in thyroid cancer. Gene expression quantification of thyroid cancer in TCGA database was extracted and divided into the “high-KCNJ16” and “low-KCNJ16” groups by the median expression. We screened 614 DEGs, with 52 upregulated genes and 562 downregulated genes. Functional and pathway enrichments were implemented (Fig. 5E). The results depicted the enriched biological processes related to cell growth and differentiation, including cell growth (GO:0016049), epithelial cell proliferation (GO:0050673), columnar/cuboidal epithelial cell differentiation (GO:0002065). Moreover, DEGs were enriched in EMT (GO:0001837) and cell-cell adhesion (GO:0022408). KEGG pathways included cell adhesion molecules and ECM-receptor interactions (GO and KEGG pathways were listed in the Additional Tables 2 and 3, respectively)[20–22].

To further explore KCNJ16 and these enriched pathway in ATC, the sample data of ATC were downloaded from the GEO database. DEGs were received from “high-KCNJ16” group when compared to “low-KCNJ16” group. KCNJ16 associated DEGs in ATC enriched in some similar pathways. And GSEA analysis was performed to observe up-regulation or down-regulation of those interested pathways (Fig. 5F). Pathways of “thyroid gland development”, “positive regulation of epithelial cell differentiation” and “negative regulation of epithelial to mesenchymal transition” were upregulated in “high-KCNJ16” group, indicating that KCNJ16 might promote differentiation and inhibit migration. Although “regulation of MAP kinase activity” pathway was up-regulated, “ERK1 and ERK2 cascade” and NF-κB signaling pathway were down-regulated. Normalized enrichment score and P-value of the interested pathways were listed in additional Table 4. These signaling pathways were involved not only in the normal physiological function and cell differentiation of the thyroid, but also in the invasive changes of thyroid cancer [23–25]. Therefore, the role of KCNJ16 and enriched pathways in thyroid cancer need to be further investigated.

Kir5.1 (encoded by KCNJ16) reportedly forms a heterotetramer with Kir4.1 (encoded by KCNJ10); however, the expression of KCNJ10 in thyroid tissues was previously lower than that of KCNJ16. KCNJ15 and KCNJ16 were highly expressed in thyroid tissues (Additional Fig. 3). We hypothesized that, Kir4.2/Kir5.1 was the functional heterotetramer in thyroid tissues, instead of the classical Kir4.1/Kir5.1. This hypothesis was supported by the observation that KCNJ10 was seldom expressed in thyroid samples, whereas KCNJ15 and KCNJ16 were synchronously expressed in every sample (Additional Fig. 4A and B). Thus, we concluded that KCNJ16 might affect the differentiation and development of thyroid cancer alone or by the Kir4.2/Kir5.1 heterotetramer.

To determine the interaction between KCNJ16 and TSHR, over-expression vectors of KCNJ16 were constructed and transfected into the PTC cell line K1, FTC cell line FTC133 and ATC cell line CAL62. In K1, FTC133 and CAL62, KCNJ16 was significantly over-expressed following transfection (Additional Fig. 4C). But no significant increase in TSHR expression was observed in K1 and FTC133 when KCNJ16 was over-expressed. Only in CAL62, we observed a nearly eight-fold increase in TSHR expression (Additional Fig. 4D). Then we used flow cytometry to detect TSHR expression on membrane. In three cell lines, membrane TSHR expression was not detected, even in CAL62 with KCNJ16 over-expression (Additional Fig. 4E).

### Using the deep docking platform and VFVS to screen ligands against Kir5.1

**Fig. 6:**
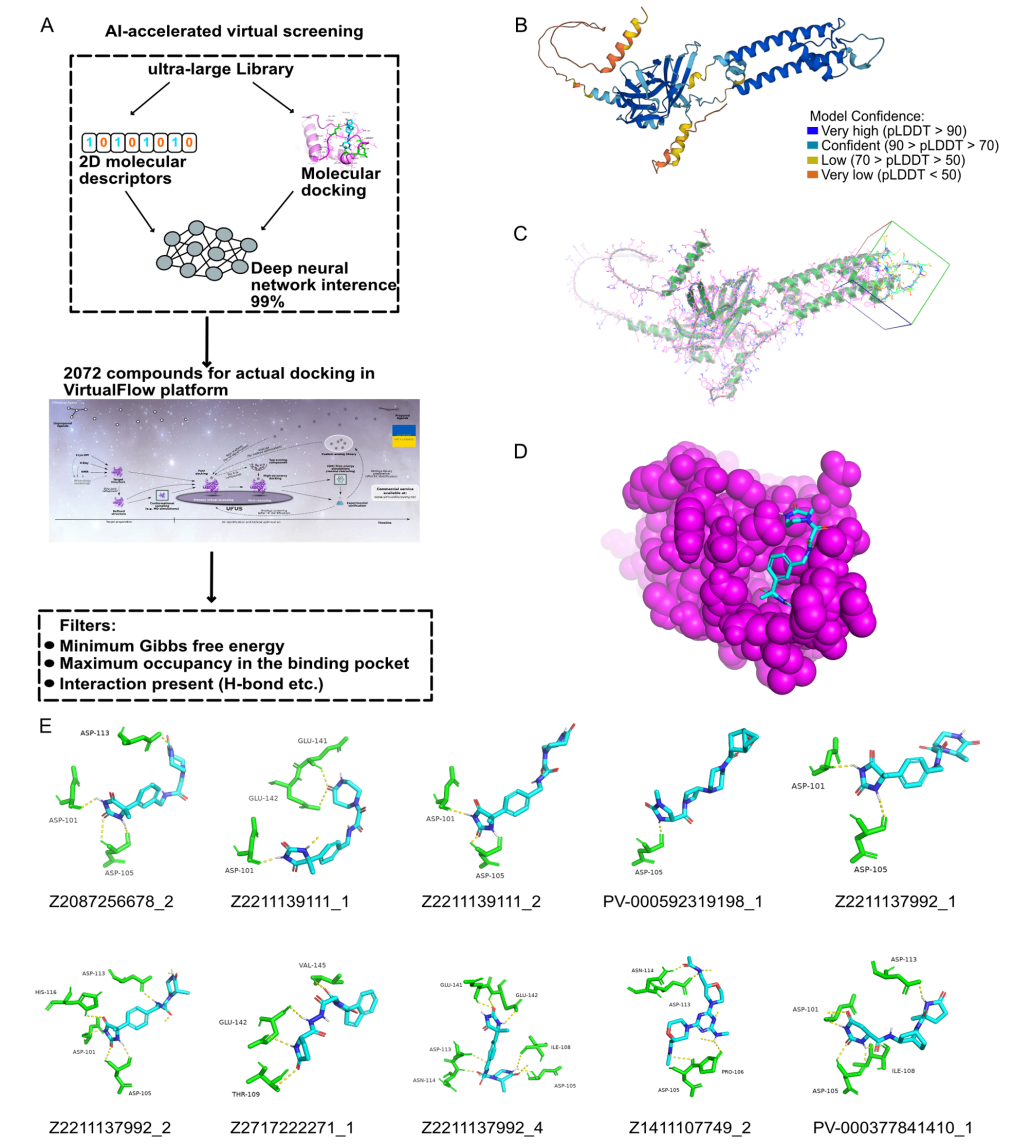
Virtual screening for ligands against Kir5.1 (**A**) A schematic of the virtual screening workflow. (**B**) Predicted crystal structure of Kir5.1. Model confidence indicates the degree of similarity with the true structure. (**C**) Visualization of the target docking position and size of Kir5.1 (inside the box), processed by Autodock Vina. (**D**) Diagram of interaction position between docking compound and Kir5.1. Structure domain in purple is the box section of Kir5.1 in (**C**). Indigo structure is the screened compound. (**E**) Docking poses of the leading 10 candidate compounds. Yellow dotted lines represent hydrogen bonds. Indigo structures are the candidate compounds. Green structures are hydrogen-bonded amino acids of Kir5.1. Kir5.1: protein encoded by KCNJ16.

First, we narrowed down a subset library of Real to approximately 1% using the deep neural network (Fig. 6A). Eventually we considered 2 072 compounds for the final docking. Every ligand tried nine poses using two scoring methods (qvina02 and smina), and we ranked the maximum affinity value. The A.I. system predicted the 3-D structure of Kir5.1 (Fig. 6B). The PDB file of Kir5.1 was processed with Autodock vina to add polar hydrogen and set the grid box for the interaction pocket (Fig. 6C). Figure 6D depicted an example of the superimposition of the docking compounds-Kir5.1 from the crystal structure. The corresponding scores of the ligands were listed in Table 3 (leading 10) and Additional Table 5 (leading 100). Consequently, we selected the leading 10 candidates to analyze the docking conformations in the best pose out of nine (Fig. 6E). Maximum candidates formed hydrogen bonds at ASP101, ASP113, and ASP105, which belong to the extracellular domain near TM1. Contrarily, hydrogen bonds usually interacted with GLU141, GLU142, and VAL145, which belong to the extracellular domain near TM2, thereby supporting the hypothesis that a series of three amino acids in this region were important for channel gating. Overall, 11 residues, including ASP113, ASP101, ASP105, GLU141, GLU142, HIS116, THR109, VAL145, ILE108, ASN114, and PRO106, were identified important for Kir5.1 binding.

**Table 3:** The top 10 ligands against Kir5.1

Rank	Ligand	score(kcal/mol)	Hydrogen bond
1	Z2087256678_2	-7.3	ASP113, ASP101, ASP105
2	Z2211139111_1	-7.3	ASP101, GLU141, GLU142
3	Z2211139111_2	-7.3	ASP101, ASP105
4	PV-000592319198_1	-7.3	ASP105
5	Z2211137992_1	-7.2	ASP101, ASP105
6	Z2211137992_2	-7.2	ASP101, ASP105, ASP113, HIS116
7	Z2717222271_1	-7.1	THR109, GLU142, VAL145
8	Z2211137992_4	-7.0	ASP105, ILE108, ASP113, ASN114, GLU141, GLU142
9	Z1411107749_2	-7.0	ASP105, PRO106, ASP113, ASN114
10	PV-000377841410_1	-6.9	ASP101, ASP105, ILE108, ASP113

## Discussion

In this study, we focused on TSHR and TSHR related DEGs, KCNJ16 and SYT1. In thyroid cancer, TSHR expression was reduced in primary tumor and in any stages or histological subtypes, indicating its role as a biomarker. The two DEGs showed opposite tendency in the regulation of gene expression in thyroid cancer. The mRNA expression level of SYT1 was higher in tumor tissues, while the expression of KCNJ16 was positively correlated with that of TSHR and was decreased in tumor tissues. TSHR and KCNJ16 demonstrated a relationship with capsular/vascular invasion, and they showed similar function in immune infiltration. Furthermore, GO and KEGG enrichment analyses demonstrated that KCNJ16 may play a significant role in cell growth and differentiation, EMT, MAPK signaling pathway and NF-κB signaling pathway. Targeting Kir5.1 (encoded by KCNJ16), Z2087256678_2, Z2211139111_1, Z2211139111_2, and PV-000592319198_1 (-7.3 kcal/mol) were identified as the most potent commercially available molecular. Other molecular namely, Z2211137992_1, Z2211137992_2 (-7.2 kcal/mol), Z2717222271_1 (-7.1 kcal/mol), Z2211137992_4 (-7.0 kcal/mol), Z1411107749_2 (-7.0 kcal/mol), and PV-000377841410_1 (-6.9 kcal/mol) also revealed potent interactions with Kir5.1. Herein, we reported on Kir5.1 as a potential biomarker for more invasive thyroid cancer, and redifferentiation therapeutic targets for recurrence/metastases or PDTC and ATC patients. And we successfully developed and characterized 10 potent Kir5.1 interaction compounds through AI-assisted virtual screening.

TSHR is differentially expressed in the thyroid cancer cells from normal tissues or benign diseases [26–28]. Generally, our results were in accordance with other reports stating TSHR was expressed at lower levels in cancer tissues. A prior study reported that approximately half of the samples (44.2%) presented a lower staining intensity in lymph node metastases than that in their corresponding primary tumors [29]. However, our results revealed that lymph node metastases did not affect TSHR expression in the primary sites, thus indicating its expression was heterogeneously distributed. Recently, thyrocytes from subcutaneous loci belonging to distant metastases in PTC displayed lower degrees of differentiation (assessed by the TDS score, which considers TSHR expression as one of the indicators) and preferentially upregulated dedifferentiation-related transcription factors and pathways. Unsurprisingly, these samples were obtained from patients who had progressed to the RAI-refractory (RAIR) status [30]. Furthermore, the normal expression and function of TSHR was essential for the character of thyroid cancer cells and the prognosis of patients with an RAIR status.

Potassium channel regulation may influence tumor progression via multiple pathways, such as cell adhesion or migration, angiogenesis and apoptosis. Most Kir subunits are overexpressed in multiple types of cancers. Kir2.1 (KCNJ2) was overexpressed in 44.23% of small-cell lung cancer (SCLC) tissues and is correlated with the clinical stage and chemotherapy response in patients with SCLC [31]. Kir3.1 was another protein associated with cancer, overexpressed in non-small cell lung cancers (NSCLCs), 40% of primary human breast cancers, and breast cancer cell lines [32, 33]. A functional and pathway enrichment analysis revealed that KCNJ16 was associated with epithelial cell proliferation and differentiation, thyroid hormone generation and metabolism, epithelial to mesenchymal transition, and cell-cell adhesion. In other words, KCNJ16 may be a regulator of thyroid differentiation and cancer migration. Consistent with our findings, KCNJ16 has been reported in other cancers. Its downregulation was observed in a hepatocellular carcinoma group and PDAC [34]. And from the Human Protein Atlas, the protein expression score of KCNJ16 was similar in thyroid gland and kidney. Interesting thing was that KCNJ16 was a favorable prognostic marker in renal cancer and thyroid cancer (data not shown). KCNJ16 had the diagnostic, prognostic, and therapeutic value in clear cell renal cell carcinoma [35]. Thus, KCNJ16 may play a protective role in cell differentiation and cancer progression.

KCNJ16 expression was positively correlated with that of TSHR in our study. Particularly, the expression significantly declined in patients with vascular/capsular invasion. Moreover, the over-expression of KCNJ16 upregulated the expression of TSHR in ATC cell line CAL62. But the CT value of TSHR mRNA expression was over 30, indicating its low expression. In sharp contrast to the ubiquitous expression of TSHR in DTC clinical samples, TSHR expression appears to be absent in most DTC-derived cell lines [36]. Many literatures have reported that thyroid cancer cell lines lost TSHR expression. The absence of this protein in commonly used DTC cell lines could be attributed to gene mutation and culture-specific selective pressures in vitro and has been well documented in the literature [37–39]. And in our study, we did not detect TSHR membrane expression in cell lines, even in CAL62 with escalated TSHR mRNA expression. Both low mRNA expression and complex translation process from RNA to protein could result in no-detection. The regulation role of KCNJ16 will need more investigation in primary thyroid cell, cause cell lines have lost most differentiation features.

The complex regulatory mechanisms of Kir5.1 in cancer progression remain largely unknown. In a previous study, the potassium channel subunits, namely KCNQ1 and KCNE2, formed a thyroid-stimulating hormone-stimulated, constitutively active, thyrocyte K + channel. KCNQ1 and KCNE2 are required for normal thyroid hormone biosynthesis, and this K + voltage affects the NIS [40, 41]. So according to KCNQ1-KCNE2 K + channel was necessary for iodine uptake, the Kir may also play the similarly role in thyroid tissues. However, this hypothesis requires further investigation. The Kir4.1, Kir4.2, Kir5.1 mRNA levels had significantly increased during mouse thyroid gland development [42]. Targeting Kir5.1 may be a promising strategy for patients with an RAIR status. Samantha et al. performed a novel study comprising a wet-bench high-throughput screening of 80 475 compounds as the potent inhibitors of Kir4.1/kir5.1 heterotetramer [43]. On an average, a novel potent drug costs billions of dollars and takes more than decades to develop, particularly comprising time-consuming wet-bench experiments. Therefore, computer-assisted drug discovery can reduce the cost of ultra-large screening.

Our study had some limitations. First, our sample capacity was insufficient, specifically owing to the lack of DeTC and ATC specimens. We could not confirm DEGs expression in DeTC and ATC, despite considering ATC while selecting GEO datasets. Moreover, we verified the expression of DEGs by RT-PCR, which could only reflect the relative quantification of DEGs, but not their location or distribution in cells, thus necessitating further studies. In addition, we explored the possible mechanisms between the targeted genes using the GO and KEGG enrichment analysis. The role of these genes in thyroid differentiation and cancer progression requires further verification. Even with the accelerated A.I., our initial library contained only approximately 200 000 compounds. Our library was relatively small than the largest commercial library, i.e., Real comprising 1.3 billion compounds. While A.I. is a promising tool for identifying potential derivatives of a target and improving drug development efficiency, the actual effects of drugs and their applicability to various diseases must be evaluated through basic and clinical trials. Therefore, the conclusions of this study can only be considered suggestive, and generalization significance is lacking. Validation of the candidates from our screening through wet-bench experiments is still necessary.

In summary, we identified five common DEGs related to TSHR mRNA expression in PTC and ATC using an integrated microarray analysis. TSHR expression was closely associated with the clinicopathological characteristics, particularly the age, historical type, stage, and invasion status. KCNJ16 expression was consistent with TSHR expression, and KCNJ16 may affect thyroid differentiation and malignant transformation by regulating TSHR expression. A.I. assistant virtual screening demonstrated that Z2087256678_2, Z2211139111_1, Z2211139111_2, PV-000592319198_1, Z2211137992_1, Z2211137992_2, Z2717222271_1, Z2211137992_4, Z1411107749_2, and PV-000377841410_1 had potent interactions with Kir5.1. This study may provide greater insights into the dedifferentiation features associated with TSHR expression in thyroid cancer and Kir5.1, which may be a potential therapeutic target in redifferentiation strategies for recurrent and metastatic thyroid cancer, thus enabling the adoption of RAI therapy and other treatments.

## Electronic supplementary material

Below is the link to the electronic supplementary material.

Supplementary Material 1

Supplementary Material 2

## Data Availability

The datasets used and/or analysed during the current study are available from the corresponding author on reasonable request.

## List of Abbreviations

TSHR: Thyroid stimulating hormone receptor
TC: Thyroid cancer
PTC: Papillary thyroid carcinoma
FTC: Follicular thyroid carcinoma
ATC: Anaplastic thyroid carcinoma
WDTC: Well-differentiated thyroid cancers
DeTCs: Dedifferentiated thyroid cancers
RAI: Radioactive iodine
NIS: Sodium/iodide symporter
TG: Thyroglobulin
TPO: Thyroperoxidase
RAIR: Resistance to RAI therapy
EMT: Epithelial-mesenchymal transition
TCGA: The Cancer Genome Atlas
GEO: Gene Expression Omnibus
DEGs: Differentially expressed genes
MTC: Medullary thyroid carcinoma
GO: Gene Ontology
KEGG: Kyoto Encyclopedia of Genes and Genomes
