# Supplementary material for: Targeting the inward rectifier potassium channel 5.1 in thyroid cancer: artificial intelligence-facilitated molecular docking for drug discovery
Source: BMC Endocr Disord. 2023 May 19;23:113. doi: 10.1186/s12902-023-01360-z (PMC10197823; doi:10.1186/s12902-023-01360-z)
Supplement: Supplementary file 2 — Supplementary Material 2 [file 12902_2023_1360_MOESM2_ESM.pdf]

Additional file 1: the sequence of KCNJ16-CDS

ATGAGCTATTACGGCAGCAGCTATCATATTATCAATGCGGACGCAAAATACCCAGGCTACCCGCC
AGAGCACATTATAGCTGAGAAGAGAAGAGCAAGAAGACGATTACTTCACAAAGATGGCAGCTG
TAATGTCTACTTCAAGCACATTTTTGGAGAATGGGGAAGCTATGTGGTTGACATCTTCACCACTC
TTGTGGACACCAAGTGGCGCCATATGTTTGTGATATTTTCTTTATCTTATATTCTCTCGTGGTTGAT
ATTTGGCTCTGTCTTTTGGCTCATAGCCTTTCATCATGGCGATCTATTAAATGATCCAGACATCACA
CCTTGTGTTGACAACGTCCATTCTTTCACAGGGGCCTTTTTGTTCTCCCTAGAGACCCAAACCAC
CATAGGATATGGTTATCGCTGTGTTACTGAAGAATGTTCTGTGGCCGTGCTCATGGTGATCCTCCA
GTCCATCTTAAGTTGCATCATAAATACCTTTATCATTGGAGCTGCCTTGGCCAAAATGGCAACTGC
TCGAAAGAGAGCCCAAACCATTCGTTTCAGCTACTTTGCACTTATAGGTATGAGAGATGGGAAG
CTTTGCCTCATGTGGCGCATTGGTGATTTTCGGCCAAACCACGTGGTAGAAGGAACAGTTAGAG
CCCAACTTCTCCGCTATACAGAAGACAGTGAAGGGAGGATGACGATGGCATTTAAAGACCTCAA
ATTAGTCAACGACCAAATCATCCTGGTCACCCCGGTAACTATTGTCCATGAAATTGACCATGAGA
GCCCTCTGTATGCCCTTGACCGCAAAGCAGTAGCCAAAGATAACTTTGAGATTTTGGTGACATTT
ATCTATACTGGTGATTCCACTGGAACATCTCACCAATCTAGAAGCTCCTATGTTCCCCGAGAAATT
CTCTGGGGCCATAGGTTTAATGATGTCTTGGAAGTTAAGAGGAAGTATTACAAAGTGAACTGCTT
ACAGTTTGAAGGAAGTGTGGAAGTATATGCCCCCTTTTGCAGTGCCAAGCAATTGGACTGGAAA
GACCAGCAGCTCCACATAGAAAAAGCACCACCAGTTCGAGAATCCTGCACGTCGGACACCAAG
GCGAGACGAAGGTCATTTAGTGCAGTTGCCATTGTCAGCAGCTGTGAAAACCCTGAGGAGACC
ACCACTTCCGCCACACATGAATATAGGGAAACACCTTATCAGAAAGCTCTCCTGACTTTAAACAG
AATCTCTGTAGAATCCCAAATGTAG

Additional Fig 1: The DNA methylation of KCNJ16 in thyroid cancer

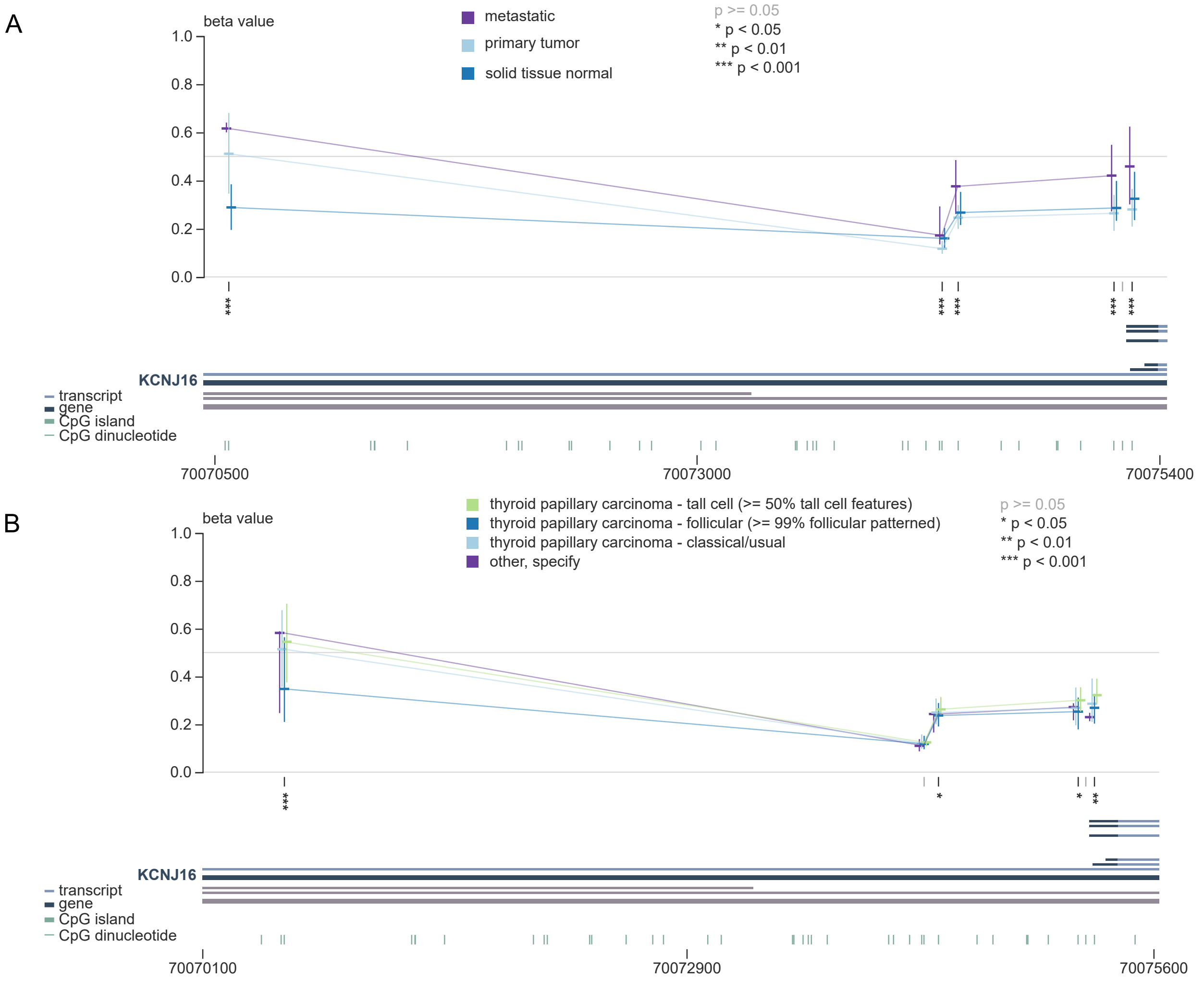

A. The status of DNA methylation in sample types of thyroid cancer in MEXPRESS. B. The status of DNA methylation in histological subtypes of thyroid cancer in MEXPRESS. Beta value indicates the degree of methylation.

Additional Fig 2: The immune cell infiltration of target genes in thyroid cancer

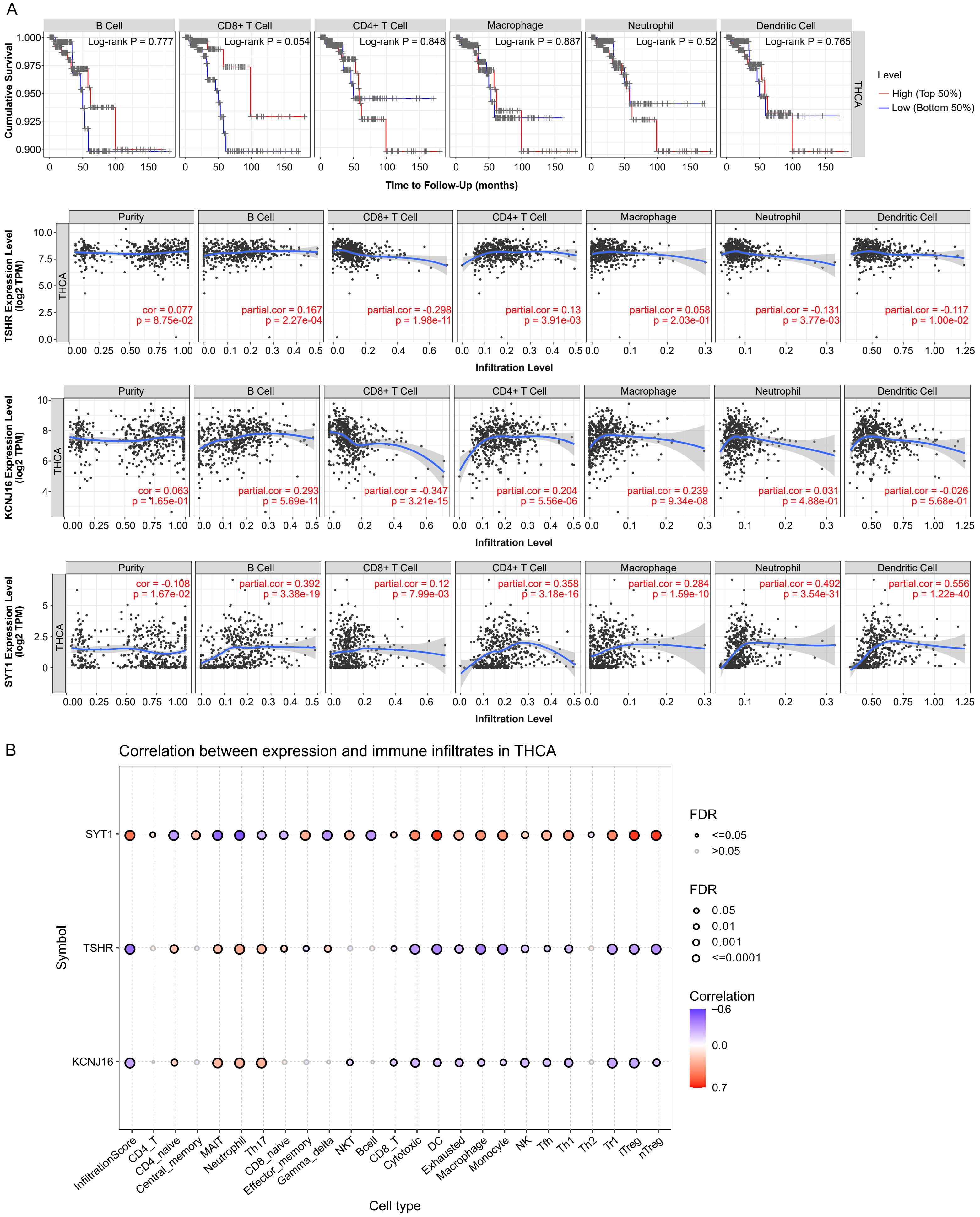

A. Immune infiltration of TSHR, KCNJ16 and SYT1 in TIMER database. B. The correlation between expression of TSHR, KCNJ16, SYT1 and immune infiltrates in THCA from GSCA database. THCA: thyroid cancer.

Additional Fig 3: KCNJ15 and KCNJ16 RNA tissue specificity

A

KCNJ15 RNA tissue specificity: Tissue enhanced (kidney, thyroid gland)

Consensus dataset

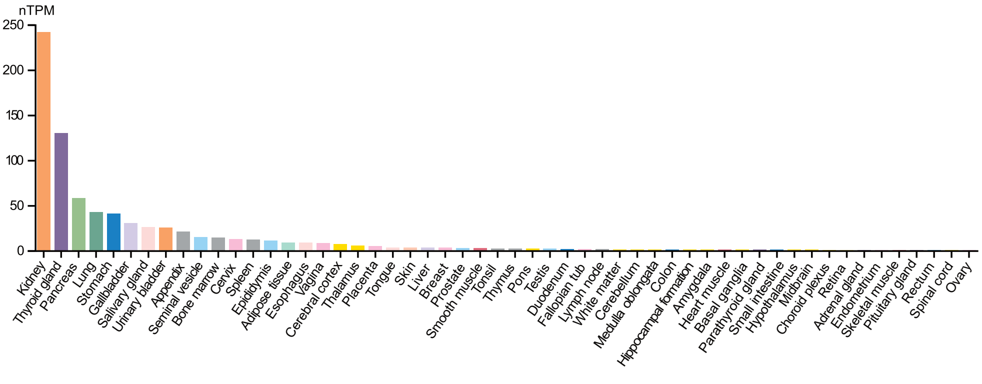

HPA dataset

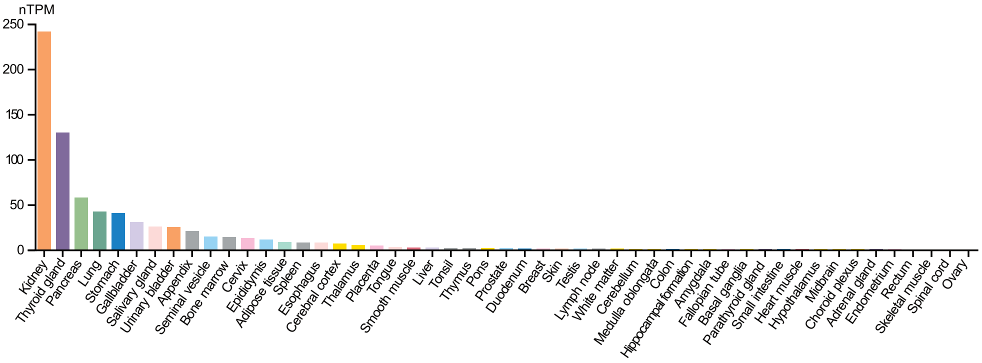

B

KCNJ16 RNA tissue specificity: Group enriched (kidney, parathyroid gland, thyroid gland)

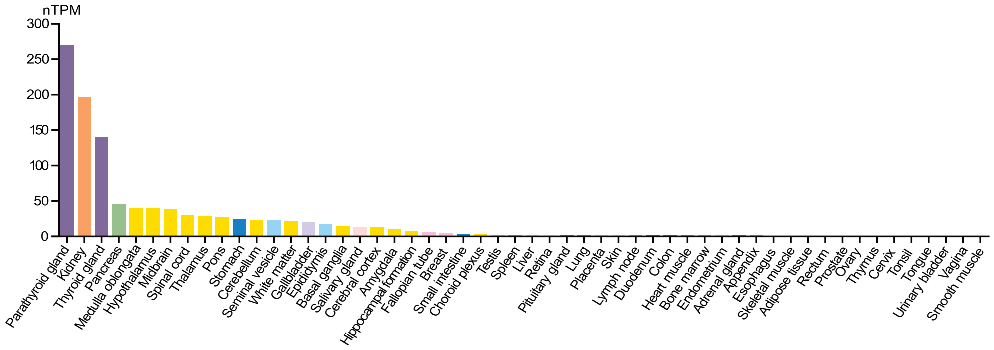

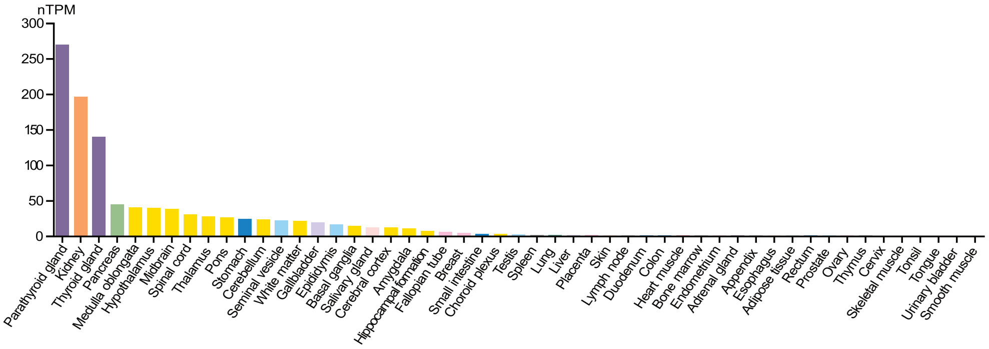

Additional Fig 4:Regulation of KCNJ16 expression in thyroid cancer cell lines

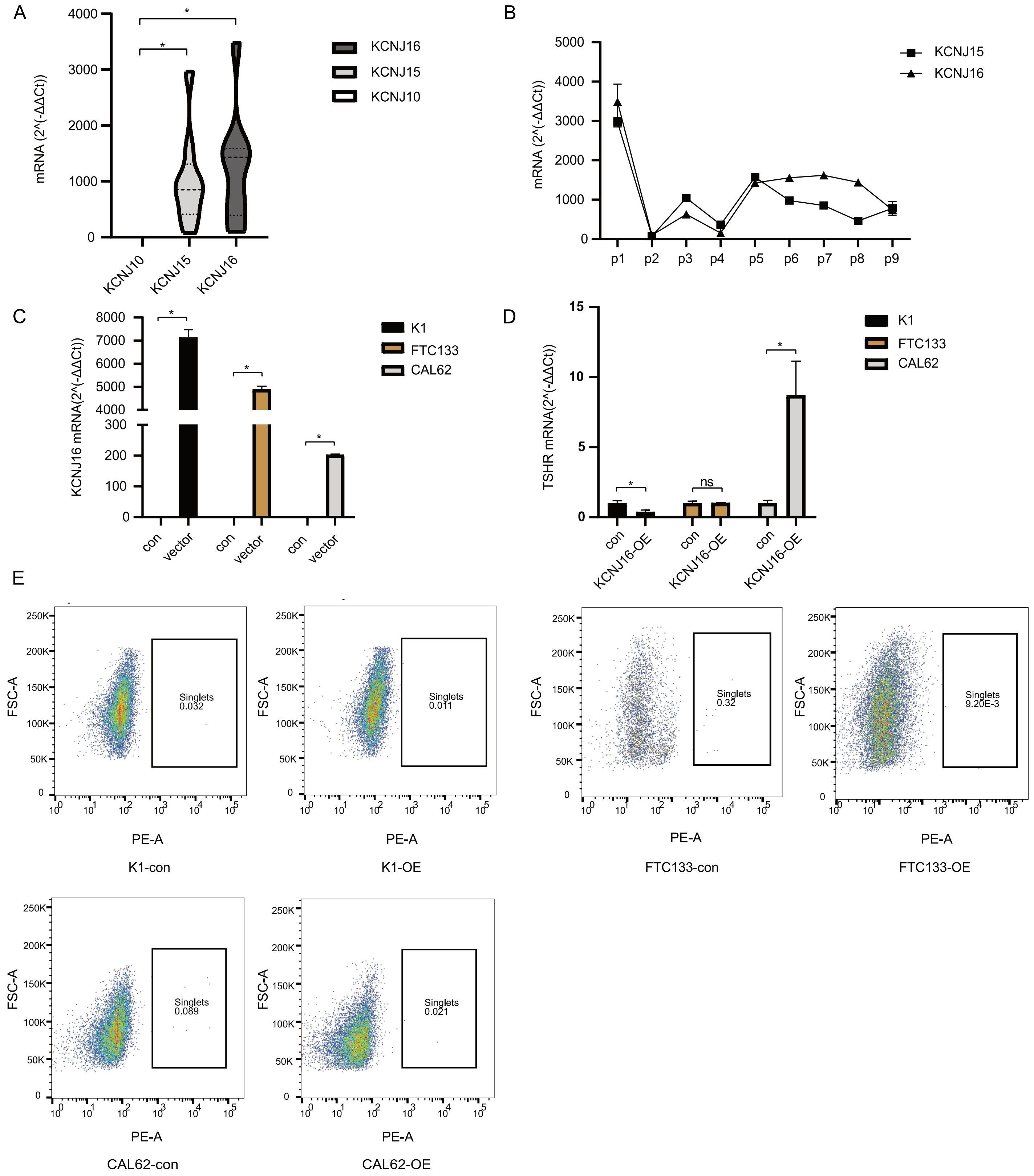

A. The mRNA expression of KCNJ10, KCNJ15 and KCNJ16 in nine samples with thyroid cancer. B. The expression level of KCNJ10 and KCNJ16 in the same sample. C. KCNJ16 expression after over-expression vector of KCNJ16 was transfected in thyroid cancer cell lines K1, FTC133 and CAL62. D. TSHR expression between control group (con) and KCNJ16-overexpression group (KCNJ16-OE) in K1, FTC133 and CAL62. E. TSHR membrane expression between control group (con) and KCNJ16 overexpression group (OE) in K1, FTC133 and CAL62. *: $P < 0.05$.
